# Bone Marrow Aspirate Concentrate Combined with Ultra-Purified Alginate Bioresorbable Gel Enhances Intervertebral Disc Repair in a Canine Model: A Preclinical Proof-of-Concept Study

**DOI:** 10.3390/cells13110987

**Published:** 2024-06-05

**Authors:** Daisuke Ukeba, Yoko Ishikawa, Katsuhisa Yamada, Takashi Ohnishi, Hiroyuki Tachi, Khin Khin Tha, Norimasa Iwasaki, Hideki Sudo

**Affiliations:** 1 Department of Orthopedic Surgery, Hokkaido University Hospital, N14W5, Sapporo, Hokkaido 060-8638, Japan; daisuke922@nifty.com (D.U.); y.ishikawa1105@gmail.com (Y.I.); yka2q@pop.med.hokudai.ac.jp (K.Y.); takashi.onishi.ortho@gmail.com (T.O.); hitachi198885@gmail.com (H.T.); niwasaki@med.hokudai.ac.jp (N.I.); 2 Laboratory for Biomarker Imaging Science, Graduate School of Biomedical Science and Engineering, Hokkaido University, N15 W7, Kita-ku, Sapporo 060-8638, Japan; kktha@med.hokudai.ac.jp

**Keywords:** low back pain, intervertebral disc herniation, intervertebral disc regeneration, bone marrow aspirate concentrate, ultra-purified alginate

## Abstract

Although discectomy is commonly performed for lumbar intervertebral disc (IVD) herniation, the capacity for tissue repair after surgery is limited, resulting in residual lower back pain, recurrence of IVD herniation, and progression of IVD degeneration. Cell-based therapies, as one-step procedures, are desirable for enhancing IVD repair. This study aimed to investigate the therapeutic efficacy of a combination of newly developed ultra-purified alginate (UPAL) gel and bone marrow aspirate concentrate (BMAC) implantation for IVD repair after discectomy. Prior to an in vivo study, the cell concentration abilities of three commercially available preparation kits for creating the BMAC were compared by measuring the number of bone marrow mesenchymal stem cells harvested from the bone marrow of rabbits. Subsequently, canine-derived BMAC was tested in a canine model using a kit which had the highest concentration rate. At 24 weeks after implantation, we evaluated the changes in the magnetic resonance imaging (MRI) signals as well as histological degeneration grade and immunohistochemical analysis results for type II and type I collagen-positive cells in the treated IVDs. In all quantitative evaluations, such as MRI and histological and immunohistochemical analyses of IVD degeneration, BMAC-UPAL implantation significantly suppressed the progression of IVD degeneration compared to discectomy and UPAL alone. This preclinical proof-of-concept study demonstrated the potential efficacy of BMAC-UPAL gel as a therapeutic strategy for implementation after discectomy, which was superior to UPAL and discectomy alone in terms of tissue repair and regenerative potential.

## 1. Introduction

Lumbar intervertebral disc (IVD) herniation is a common spinal disease, with discectomy used as the current surgical treatment. However, discectomy is associated with a number of limitations, such as limited natural repair of the IVD after treatment, residual lower back pain, recurrence of IVD herniation, and progression of IVD degeneration [1,2]. Bone marrow-derived mesenchymal stem cell (BMSC) transplantation may represent a valid measure for the treatment of degenerated IVD disease [3,4].

In addition, bone marrow aspirate concentrate (BMAC) has recently attracted attention as a useful treatment for osteoarthritis [5] and ligament injury [6]. There are also clinical research reports on the subcutaneous administration of BMAC to treat discogenic lower back pain [7,8]. Administration of BMAC is a one-step cell-based technique, and the utilization of autologous, non-cultured cells reduces the risk of infection and sample confusion compared with culture-expanded cells [7,8,9].

However, when cells are used to treat a herniated disc, the transplanted cells can easily flow out owing to intradiscal pressure. There are no clinically available biomaterials applicable to discectomy-associated IVD defects [9]. Tsujimoto et al. [2] successfully developed a bioresorbable ultra-purified alginate (UPAL) gel to prevent post-discectomy IVD degeneration in rabbit and sheep models. The material could gelate within 5 min in situ and exhibited sufficient biomechanical properties without material protrusion after discectomy. CaCl_2_ surface coverage was used for alginate gelation without the need for suturing of the annulus fibrosis (AF). In addition, the UPAL gel has high purity and low endotoxicity (<1/10,000 compared to commercially available laboratory alginate), making it suitable for clinical application by preventing immunologic reactions to the implanted material [2,9]. Clinical research is currently underway to implant this biomaterial into the IVD cavity after discectomy [10].

Ukeba et al. [9] investigated the repair efficacy of BMAC-UPAL gels in the treatment of IVD defects after discectomy in rabbits. Significant histological improvements were observed in the BMAC-UPAL group compared with those treated with either UPAL gel or discectomy. The study also demonstrated that the mechanical stability of BMAC embedded in UPAL gel did not alter the mechanical characteristics of the gel [9]. These results indicated that BMAC enhances the repair of IVD defects after discectomy and may be applicable in a large animal model. 

Neither commercially available preparation kit is guaranteed for use on anything other than rabbits, dogs, or humans by the corporations. Furthermore, as far as we have been able to research in the literature, there is no evidence of its use in large animals such as sheep and pigs for the purpose of administering it to the IVD, with the aim of examining its clinical application. This study aimed to assess the effectiveness of BMAC-UPAL implantation after discectomy in beagle dogs as a larger animal model to confirm its potential use in future clinical applications.

## 2. Materials and Methods

### 2.1. In Vitro Study

#### 2.1.1. Animal Experimentation for In Vitro Study

Prior to an in vivo study, the cell concentration abilities for creating the BMAC were compared among three commercially available preparation kits (Bio CUE, Zimmer Biomet Holdings, Inc., Warsaw, IN, USA; SmartPrep, Terumo BCT Japan, Inc., Tokyo, Japan; and Condensia, KYOCERA Co., Kyoto, Japan). The concentration capacity was calculated by measuring the number of BMSCs harvested from the bone marrow (BM) of Japanese white rabbits. Rabbits were used instead of dogs because rabbits are more readily available than dogs and have been used in previous experiments [7]. All animal procedures were approved by the Institutional Animal Care and Use Committee of Hokkaido University (17-0122) and performed in accordance with the approved guidelines. We obtained 12 rabbits (20-week-old male Japanese white rabbits, 3.2–3.5 kg) from Sankyo Labo Service Corporation (Tokyo, Japan) for the in vitro study.

#### 2.1.2. Preparation of BMAC

Under general anesthesia, with an intravenous injection of ketamine (10 mg/kg), xylazine (3 mg/kg), and O_2_ and air (3.0 L/min) mixed with sevoflurane (2–3%) in spontaneous ventilation, 20 mL of BM (mixed with 10 mL of heparin) was collected from the iliac crests of the rabbits using 18-gauge (G) needles with a small incision (5 mm). Approximately 2 mL of BMAC was obtained by centrifugation, which was performed in accordance with the manufacturer’s instructions. 

#### 2.1.3. Measuring Cell Counts and Calculation of Concentration Ratio

First, red blood cell (RBC) lysis buffer (Takara Bio Inc., Shiga, Japan) was used to hemolyze the RBCs to obtain BM (non-concentrated): 1 mL and BMAC: 1 mL, respectively. Immunostaining was performed using CD44 (Anti-CD44 Rat-Mono (Hermes-1) FITC; Novus Biologicals, Cat# NBP2-22530F, Centennial, CO, USA), a positive marker, and CD45 (Anti-CD45 Polyclonal Antibody, Cy5.5 Conjugated; Bioss Inc., Cat# bs-0522R-Cy5.5, Woburn, MA, USA), a negative marker in rabbit stem cells [11,12]. CD44-positive and CD45-negative cells were counted using a BD FACSAria III high-speed cell sorter with Diva software version 7.0 (BD Biosciences, San Jose, CA, USA). The concentration ratio of each kit was calculated based on the number of CD44-positive and CD45-negative cells per unit volume of the BM and BMAC (*n* = 4). The purpose of this experiment was not to strictly characterize BMSCs, but to estimate the concentration ability of CD44-positive and CD45-negative cells. In addition, the percentage of CD44-positive and CD45-negative cells among the total cells was not measured because measuring the number of the cells was the main purpose of this investigation.

### 2.2. In Vivo Study

#### 2.2.1. Animal Experimentation for In Vivo Study

For use in future clinical applications, we aimed to assess the effectiveness of BMAC and UPAL gel implantation after discectomy in a large animal model of beagle dogs. In this in vivo study, all procedures were performed in a medical product Good Laboratory Practice (GLP)-adapted laboratory (Hamri Co., Ltd., Ibaraki, Japan) [2,13], and we obtained seven male beagle dogs (19–22 months old, weighing 11.5–12.5 kg) for the experiments. A total of 28 IVDs (L1/2, L2/3, L3/4, and L4/5) were randomly allocated to the intact control (*n* = 6), discectomy (*n* = 6), UPAL (*n* = 8), and BMAC-UPAL (*n* = 8) groups [2,9,13,14,15,16,17,18,19]. Although it is rare for IVD herniation to occur in several spots simultaneously, this model was adopted to reduce the number of experimental animals. The abortion criteria of these experiments were postoperative complications such as surgical site infection. However, there were no complications or drop-outs during the animal experiments.

#### 2.2.2. Collecting BM and Preparation of BMAC

**Figure 1:**
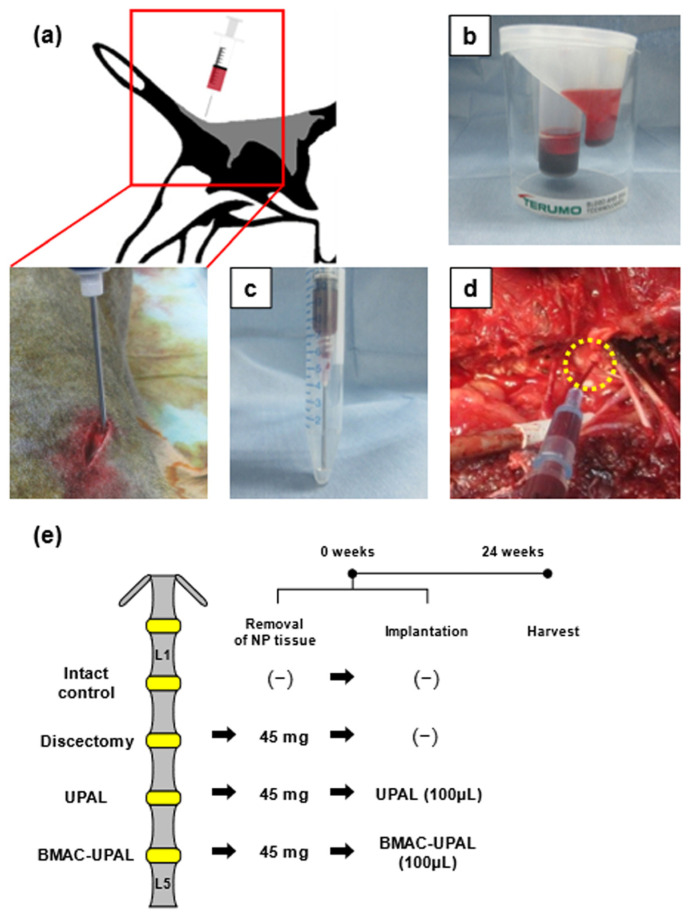
Schematic of the experimental process and time schedule from bone marrow (BM) collection to material implantation. We obtained 7 male beagle dogs (19–22 months old) for the experiments. (**a**) Under general anesthesia, 20 mL of BM was collected from the iliac crest at the start of the operation. (**b**) Using the bone marrow aspirate concentrate (BMAC) preparation kit (SmartPrep), BMAC was obtained by centrifugation. (**c**) Preparation of BMAC and ultra-purified alginate (UPAL) mixture. (**d**) After discectomy, the intervertebral disc (IVD) cavity was filled with the BMAC-UPAL mixture or UPAL solution. (**e**) Time schedule and treatment details for each treatment group.

In this in vivo study, we used the BM from beagles to perform the experiment, and we decided to use SmartPrep, which had the highest concentration rate among the three kits, for BMAC preparation, as shown in the results bellow. Beagle dogs were intramuscularly administered general anesthesia with ketamine (20 mg/kg), xylazine (8 mg/kg), and O_2_ mixed with isoflurane (2–3%) in spontaneous ventilation. First, 25 mL of BM was collected from the iliac crest of the dogs using 15 G intraosseous needles (BMHN1502; SHEEN MAN Co., Ltd., Osaka, Japan) and mixed with 5 mL of heparin as an anticoagulant before IVD treatment (Figure 1a). A total of 30 mL of anticoagulated BM was transferred to a SmartPrep kit and centrifuged at 2500 rpm for 14 min (Figure 1b). Finally, 2–3 mL of BMAC was obtained. In addition, 1 mL of BMAC was mixed with 1 mL of 4% (*w*/*v*) UPAL (Sea Matrix; Mochida Pharmaceutical Co. Ltd., Tokyo, Japan) solution to prepare a BMAC-UPAL mixture (2%) for implantation [9] (Figure 1c). UPAL was dissolved in phosphate-buffered saline (PBS; Wako Pure Chemical Industries, Osaka, Japan) before use, and the mechanical safety of the 2% UPAL gel was demonstrated as previously described [9,13,20].

#### 2.2.3. Discectomy and BMAC-UPAL Implantation

After preparing the BMAC-UPAL mixture, discectomy and implantation were performed using an anterolateral retroperitoneal approach. In all IVDs, with the exception of the intact control group, approximately 40 mg (42.0 ± 3.1 mg) of nucleus pulposus (NP) tissues were removed to generate an IVD cavity using a pair of forceps following exposure. Thereafter, in the UPAL group, we filled the IVD defect with approximately 110 µL (109 ± 53 µL) of 2% UPAL solution with an 18 G needle. In contrast, in the BMAC-UPAL group, the same amount of BMAC-UPAL mixture was placed into the IVD cavity using a similar technique (Figure 1d). Lastly, a 102 mM CaCl_2_ solution was injected on top of the implanted material for gelation. After 5 min, the operative wound was washed with normal saline and closed [2,9,13,20]. The treated dogs were euthanized 24 weeks after surgery for the qualitative and/or quantitative evaluation of IVD degeneration (Figure 1e). Because we previously investigated the repair efficacy of BMAC-UPAL gels in the treatment of IVD defects after discectomy in rabbits (4 weeks and 12 weeks) and confirmed that IVD degeneration progresses over time [7], and because we wanted to reduce the number of experimental animals, the evaluation time point was limited to 24 weeks.

#### 2.2.4. Magnetic Resonance Imaging (MRI) Analysis

At 24 weeks after implantation, we evaluated the MRI signal changes in the treated IVDs. Spinal specimens (L1-sacrum) were collected after euthanasia, and T2-weighted midsagittal section images were obtained using a 3.0 T scanner (MAGNETOM Prisma; Siemens, Munich, Germany) [13]. The degree of IVD degeneration was quantified using the Pfirrmann classification [21], which categorizes IVDs according to a five grade system (1, normal; 5, severely degenerated). All image assessments were performed by three independent observers who were blinded to the samples, and the mean of the three evaluations was recorded. The MRI index values were also measured using Analyze 14.0 software (AnalyzeDirect, Overland Park, KS, USA). These values are the product of the average signal intensity of the NP and area of the NP. We calculated the relative MRI index of the target IVDs relative to the values of normal IVDs (intact control), as previously described [2,9,13,20,22,23]. We did not evaluate the disc height.

#### 2.2.5. Histological Analysis

Following MRI analysis, histological analysis was performed. IVD samples extracted from the spine were fixed in 10% formaldehyde, decalcified with 10% EDTA (pH 7.5), and embedded in paraffin. Midsagittal 5 µm thick paraffin sections were pretreated with xylene, alcohol, and water and stained with hematoxylin and eosin (H&E) and safranin-O. Because we have performed similar evaluations in previous studies [2,9,13,20,22,23], we adopted this staining method to confirm consistency and did not perform Masson’s trichrome or Picrosirius red staining. We performed semiquantitative analysis based on the histological grade, focusing on the structural collapse of the inner AF [2,9,20,24,25]. The degree of IVD degeneration was classified based on six levels, from 0 (normal) to 5 (severely degenerated), focusing on morphological changes in the AF structures, as previously reported [2,9,20,24,25]. All image assessments were performed by three independent blinded observers, and the mean of the three evaluations was recorded.

#### 2.2.6. Immunohistochemical Analysis

Immunohistochemical (IHC) analysis was also performed to evaluate the expression of type II and type I collagen in the treated IVDs, as previously described [2,9,13,20]. Type II collagen, an extracellular matrix component, is abundant in normal NP tissues, whereas type I collagen increases as IVD degeneration progresses [26]. The sections were treated with 0.1% trypsin to activate the antigens, followed by deparaffinization with xylene. After protein blocking using Protein Block Serum-Free (Dako, Agilent Technologies, Cat # X0909, Santa Clara, CA, USA), the sections were treated with 3% H_2_O_2_. Thereafter, goat anti-type I collagen (Southern Biotech, Cat# 1310-01, Birmingham, AL, USA) and anti-type II collagen mouse (Kyowa Pharma Chemical Co., Ltd., Cat# F-57, Toyama, Japan) were used as primary antibodies, while histfine simple stain max-PO(G) (Nichirei Biosciences, Cat# 414162, Tokyo, Japan) and envision + system-HRP labelled polymer anti-mouse (Dako, Cat# 4001) were used as secondary antibodies for type I and type II collagen, respectively. Finally, the sections were stained with DAB (Dako, Cat# K3468) and hematoxylin, and washed with water. The sections were observed under a microscope, five fields were randomly selected, and the number of positive cells whose outer periphery was stained with type II or I collagen was calculated [2,9,13,20]. The percentage of each positive cell for type II or I collagen was calculated [2,9,13,20].

### 2.3. Statistical Analysis

For multigroup comparisons, one-way analysis of variance (ANOVA) and the Tukey–Kramer post hoc test were performed using the JMP Pro version 16.0 software (SAS Institute, Cary, NC, USA). Sample randomization was performed, and the test samples were blinded. All data are presented as mean ±SD values. A *p*-value under 0.05 was considered statistically significant.

## 3. Results

### 3.1. Comparison of Concentration Ratios among Three Commercially Available BMAC Preparation Kits

**Figure 2:**
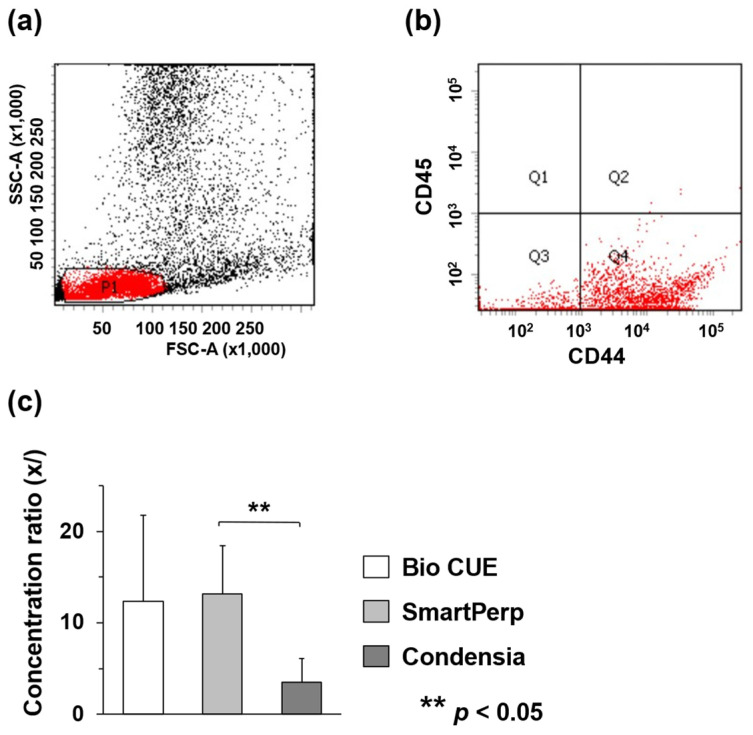
Cell sorting data on the separation and measurement of CD44-positive and CD45-negative cells from the specimens. We obtained 12 (20-week-old) male rabbits for the experiments. (**a**) Two-dimensional (2D) dot plot represents various cells including CD44-positive and CD45-negative cells and debris. The P1 gate excluded dead cells and debris, and sorted out live cells. SSC-A: Side scatter-area; FSC-A: Forward scatter-area. (**b**) The dot plot in the Q4 gate shows the CD44 (FITC)-positive and CD45 (Cy5.5)-negative cells and the number of these cells was measured. (**c**) The concentration ratio was calculated using the number of CD44-positive and CD45-negative cells per unit volume in BMAC and unconcentrated BM.

Prior to cell counting, debris and dead cells were removed using a cell sorter and various cells, including BMSCs, were obtained from the P1 gate as previously described (Figure 2a) [13,20]. In addition, CD44-positive (FITC-positive) and CD45-negative (Cy5.5-negative) cells at the Q4 gate were counted (Figure 2b). In the Bio CUE group, the number of CD44-positive and CD45-negative cells in the pre-concentration was 4.5 ± 1.1 × 10^5^/mL and that in the post-concentration was 5.1 ± 3.4 × 10^6^/mL, with a concentration ratio of 12.4-fold. In contrast, in the SmartPrep and Condensia groups, the numbers of CD44-positive and CD45-negative cells in the pre-concentration were 2.1 ± 1.5 × 10^5^/mL and 7.0 ± 2.7 × 10^5^/mL, and those in the post-concentration were 2.2 ± 0.9 × 10^6^/mL and 2.0 ± 0.6 × 10^6^/mL, respectively. The concentration ratios were 13.2-fold and 3.5-fold, respectively (Table 1). The ratio of SmartPrep was significantly higher than that of Condensia, whereas those of Bio Cue and SmartPrep tended to be higher but were not significantly different (Figure 2c).

### 3.2. BMAC-UPAL Implantation Suppressed IVD Degeneration

**Figure 3:**
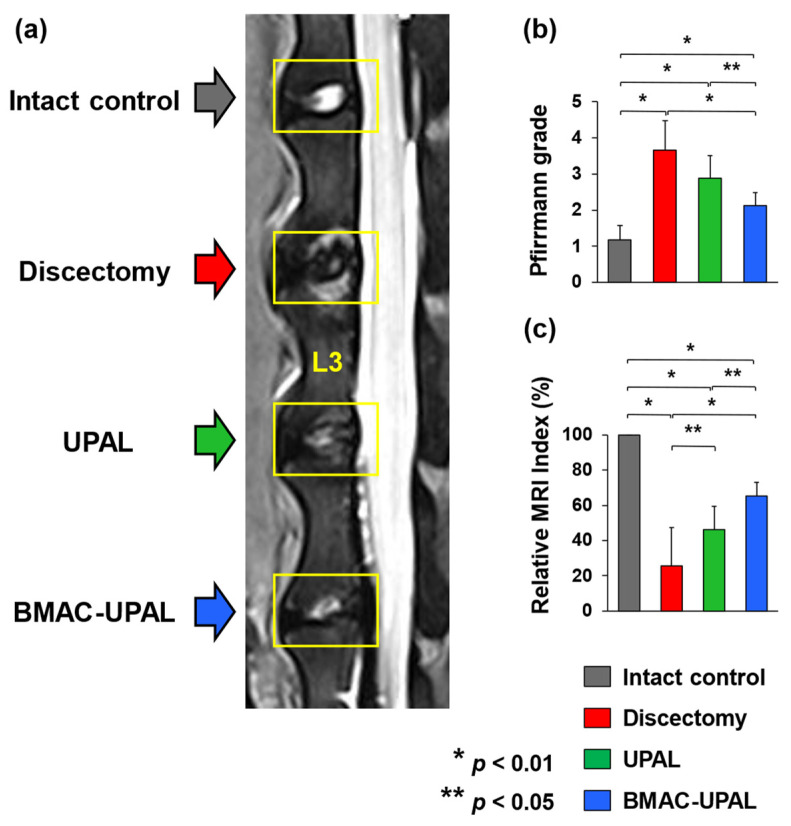
Magnetic resonance imaging (MRI) evaluation of the treated canine IVDs at 24 weeks after implantation. (**a**) T2-weighted midsagittal images of IVDs at 24 weeks after operation. (**b**) Pfirrmann grades of IVD degeneration in the four groups. (**c**) Relative MRI index (Nucleus pulposus (NP) area × average signal intensity) values indicating the degree of degenerative alterations in the NP in four groups. Numerical values are expressed as percentages relative to the values of the intact control IVDs. All data represent mean ±SD values (intact control, *n* = 6; discectomy, *n* = 6; UPAL, *n* = 8; BMAC-UPAL, *n* = 8). Testing for significant differences were conducted with one-way analysis of variance (ANOVA), with post hoc analysis using the Tukey–Kramer test.

To evaluate the tissue repair effect of BMAC-UPAL implantation on IVDs, a quantitative assessment of IVD degeneration was performed using MRI, histology, and IHC. MRI T2-weighted midsagittal images were used to evaluate signal changes in the treated IVDs (Figure 3a). Based on the Pfirrmann scores, the grades of the BMAC-UPAL group (2.13 ± 0.35) were significantly lower than those of the discectomy (3.67 ± 0.82; *p* < 0.001) and UPAL (2.88 ± 0.64; *p* = 0.048) groups (Figure 3b). Moreover, the relative MRI index was significantly higher in the BMAC-UPAL group (65.1 ± 7.9) when compared to those of the UPAL (46.2 ± 12.3; *p* < 0.045) and discectomy (25.7 ± 21.8; *p* < 0.001) groups. Interestingly, the index was higher in the UPAL group than in the discectomy group (*p* = 0.044) (Figure 3c).

**Figure 4:**
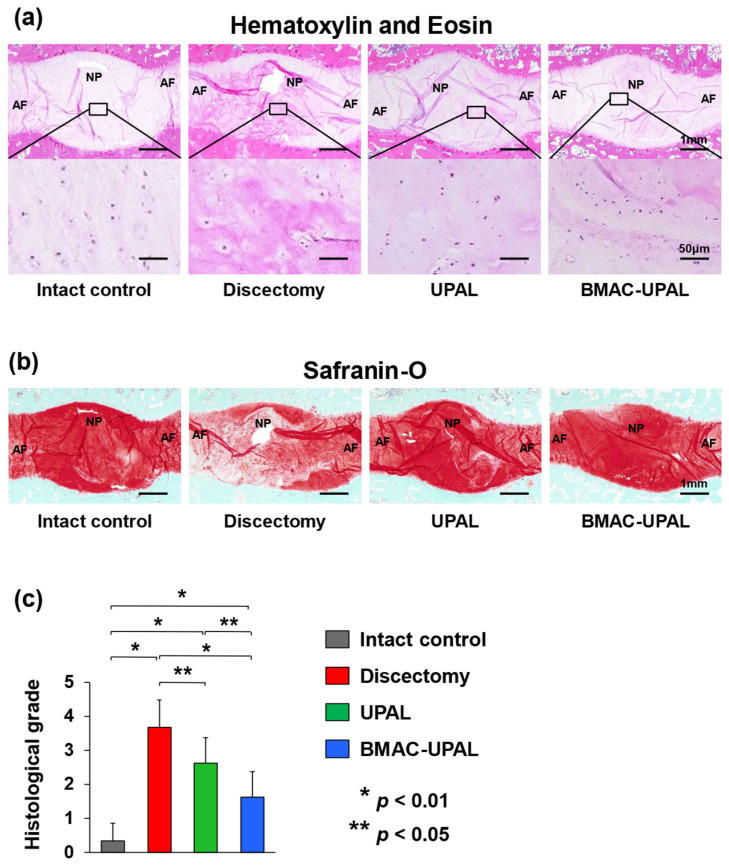
Histological evaluation of the treated IVDs at 24 weeks after operation. (**a**) Representative midsagittal sections of treated canine IVDs stained with hematoxylin and eosin in four groups. (**b**) Representative midsagittal sections of treated IVDs stained with safranin-O in four groups. Scale bar = (**a**): 1 mm (first sections) and 50 µm (second sections), (**b**): 1 mm. (**c**) Histological grades determined via previously published classifications [2,9,20,24,25]. All data represent mean ±SD values (intact control, *n* = 6; discectomy, *n* = 6; UPAL, *n* = 8; BMAC-UPAL, *n* = 8). Testing for significant differences were conducted with one-way ANOVA, with post hoc analysis using the Tukey–Kramer test. AF, annulus fibrosus; NP, nucleus pulposus.

H&E and safranin-O staining were used for histological analyses. In the intact control group, the overall morphology of the IVDs was spindle-shaped, there were no fibrotic changes in the NP tissue, the AF structures were concentric and aligned, and safranin-O staining was nearly uniform. In the BMAC-UPAL group, the IVD tissue structure was relatively preserved; however, the tissue was mildly disrupted, and fibrotic changes were partially present. Fibrotic changes in the NP and AF structure collapse were slightly more evident in the UPAL group. In the discectomy group, the IVD shape was flattened, scar tissue and fibrotic changes were evident, and some endplates were destroyed. Safranin O staining revealed an enlarged area with poor staining (Figure 4a,b).

Histological grades in the BMAC-UPAL group (1.63 ± 0.74) were significantly lower than those in the discectomy (3.67 ± 0.82; *p* < 0.001) and UPAL (2.63 ± 0.74; *p* < 0.048) groups, and those in the UPAL group were significantly lower than those in the discectomy group (*p* < 0.049) (Figure 4c).

**Figure 5:**
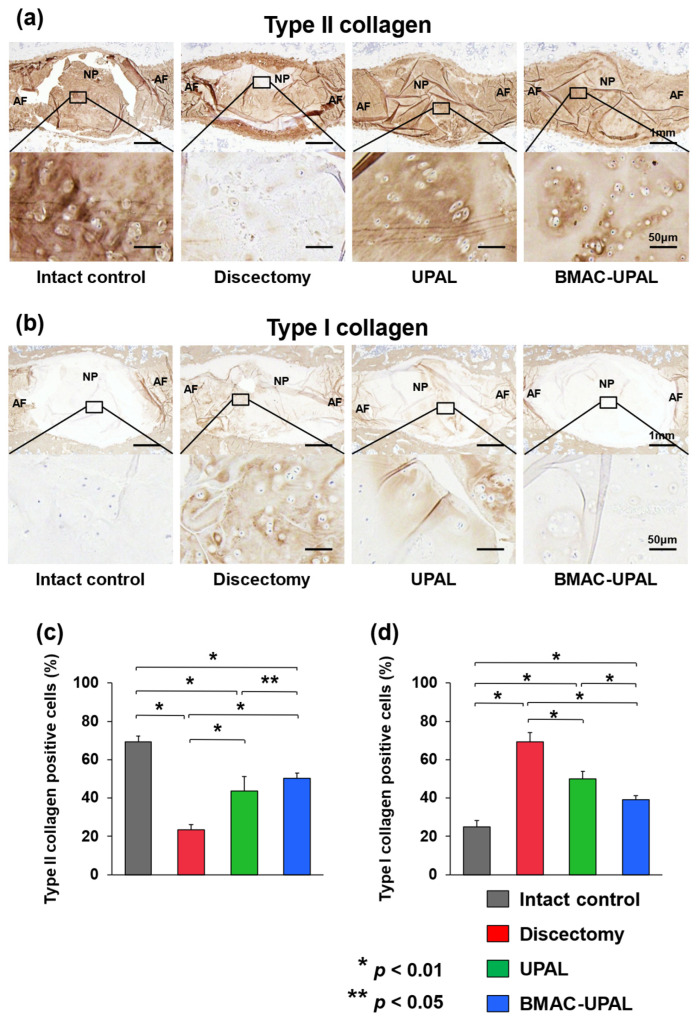
Type II or I collagen-positive canine cells in the treated canine IVDs at 24 weeks after operation. (**a**,**b**) Representative midsagittal sections of treated IVDs stained with type II collagen or type I collagen in four groups. Scale bar = (**a**,**b**): 1 mm (first sections) and 50 µm (second sections); (**c**,**d**) Percentages of Type II or I collagen-positive cells relative to total cells in the NP area of the treated IVDs. All data represent mean ±SD values (intact control, *n* = 6; discectomy, *n* = 6; UPAL, *n* = 8; BMAC-UPAL, *n* = 8). Testing for significant differences were conducted with one-way ANOVA, with post hoc analysis using the Tukey–Kramer test. AF, annulus fibrosus; NP, nucleus pulposus.

For IHC evaluation, we assessed the expression levels of type II and I collagen in the treated IVDs (Figure 5a,b). Type II collagen, an extracellular matrix component, is abundant in normal NP tissues, whereas type I collagen increases as IVD degeneration progresses [26]. The percentages of type II collagen-positive cells were significantly higher in the BMAC-UPAL group (50.4 ± 2.7) compared to the discectomy (23.5 ± 2.7; *p* < 0.001) and UPAL (43.8 ± 7.5; *p* = 0.043) groups, and those in the UPAL group were higher than those in the discectomy group (*p* < 0.001) (Figure 5c). On the other hand, the percentages of type I collagen-positive cells were significantly lower in the BMAC-UPAL (39.2 ± 2.1) group compared to the discectomy (69.2 ± 4.9; *p* < 0.001) and UPAL (49.9 ± 4.0; *p* < 0.001) groups. In addition, the percentages of type I collagen-positive cells in the UPAL group were significantly lower than that in the discectomy group (*p* < 0.001) (Figure 5d).

## 4. Discussion

In the present study, we demonstrated the reparative potential of BMAC-UPAL gel implantation in a large animal model using the GLP standard [2,13]. The BMAC-UPAL gel was significantly more effective than the UPAL gel alone in terms of reparative potential. In a previous study, UPAL gel alone promoted reparative processes in IVD tissue by facilitating the proliferation of NP progenitor cells [2]. In addition, a previous study demonstrated that the UPAL-based approach was safe and did not elicit toxic effects according to the International Organization for Standardization and GLP standards [2]. Mechanistically, BMAC is enriched with mononuclear cells, including BMSCs, and humoral factors, including growth factors [27]. Our results are understandable, given that these factors exert reparative and/or protective effects in the IVD. 

A previous study explored the role of BMSCs mounted on a UPAL gel using a sheep model [13]. Briefly, the co-culture of NP cells and BMSCs synergistically maintained the NP phenotype and boosted the production of growth factors and extracellular matrix components [13]. Moreover, in vivo implantation of the BMSC-UPAL gel drastically regenerated the IVDs after discectomy [13]. Taken together, these results suggest that the regenerative outcome was derived from phenotypic changes in NP cells and the differentiation of BMSCs through humoral factors in a reciprocal manner.

The general reparative potential of the BMAC-UPAL and BMSC-UPAL gels was comparable, although the production of type II collagen was significantly higher in the BMSC-UPAL gel group [9]. This difference may be attributed to the relatively smaller number of BMSCs contained in BMAC than in pure cultivated BMSCs [9]. However, the advantages of BMAC, which allows patients to avoid receiving allografts, are significant. As autologous blood is stored in elective surgeries, such as scoliosis surgeries in young patients [28,29], autologous material is preferable in cases of IVD regenerative therapy. This principle specifically applies to young patients with degenerative IVD who experience repetitive mechanical overload caused by sports and manifest severe degeneration of IVDs [30].

Another important aspect of cell- and biomaterial-based therapies is minimization of the risk of leakage after implantation. Leakage of the implanted cells may cause advertent osteophyte formation [31]. The stability of the UPAL gel alone after implantation was validated through rigorous biomechanical testing [2]. In addition, our previous study successfully demonstrated that the mechanical properties and stability of UPAL gel were not altered by supplementation with BMAC [9]. Collectively, the present study clarified the regenerative effects of BMAC and UPAL gel, augmenting their merits in clinical applications, in addition to the high biomechanical stability shown in our previous study [9].

In terms of clinical applications, it is necessary to consider strategies to mitigate physical and economic stresses in patients. One example of such a stress is the two-step surgery required for early cell therapy using autologous BMSCs [32,33,34]. A possible solution is to use BMAC as a replacement for autologous BMSCs to omit the first surgery. Medical costs can be reduced compared with allograft BMSCs because the preparation of BMAC is limited to BM aspiration and centrifugation [9]. Another example of stress alleviation is minimizing the risk of contamination by reducing the steps and time required to prepare the biological material. BMAC can be quickly prepared during regenerative surgery using simple aseptic procedures [6,9], possibly preventing infection of the material.

Accumulating evidence suggests that BMAC has been used in several studies, supporting its reparative capacity and safety. The vascular nature of the IVD tissue is similar to that of the articular cartilage and meniscus [35,36]. Similar to our present study, BMAC-UPAL gel was shown to repair osteochondral defects in a rabbit model [37]. BMAC itself was studied in a systematic review of knee osteoarthritis, which discussed its superiority to hyaluronic acid injection owing to its lack of adverse events [5]. BMAC was similarly evaluated for chondral or osteochondral lesions and was found to outperform microfracture or autologous chondrocyte implantation without complication [38,39,40]. Other tissues, such as ligaments and bones, were also investigated to determine the effects of BMAC. A double-blind randomized controlled trial performed to augment anterior cruciate ligament (ACL) reconstruction with BMAC indicated that ligamentization was significantly higher in the BMAC group than that after ACL reconstruction alone [6]. Bone regeneration was also confirmed in bone defects using a rabbit model [41]. Osteoinduction of hyperbaric oxygen therapy was synergistically enhanced when BMAC was used [41].

However, to date, there have been some pharmaceutical issues regarding BMAC. For example, in the United States, BMAC was approved as a medical device for concentrating BM based solely on a 510(K) Premarket Notification to the FDA [9]. This may explain the limited evidence available for this technique because the FDA does not collect elaborate preclinical or clinical data on BMAC. In the future, based on appropriate clinical trials, BMAC is expected to be approved as a medical device that guarantees clinical efficacy against certain diseases such as lumbar IVD herniation with young athletes.

This study has a few important limitations. First, although the canine models underwent discectomy using the anterolateral retroperitoneal approach, typical clinical discectomy is performed through the posterior approach [9]. Second, the composition of BMAC was not characterized [9]. The molecular mechanism related to this compound has not been elucidated. Last, we did not perform any physical examination. Canine models are not suitable for the assessment of pain-related behavior because there are still no quantitative analysis methods available. These limitations need to be carefully considered when considering the application of this research approach to humans.

## 5. Conclusions

This preclinical proof-of-concept study demonstrated the potential efficacy of BMAC-UPAL gel as a therapeutic strategy after discectomy, which was found to be superior to UPAL and discectomy alone. 

## Figures and Tables

**Table 1:** Concentration ratio in the three commercially available BMAC preparation kits.

	Number of CD44-Positive and CD45-Negative Cells Pre-Concentration (×10^5^/mL)	Number of CD44-Positive and CD45-Negative Cells Post-Concentration (×10^6^/mL)	Concentration Ratio
Bio CUE	4.5 ± 1.1	5.1 ± 3.4	12.4
SmartPrep	2.1 ± 1.5	2.2 ± 0.9	13.2
Condensia	7.0 ± 2.7	2.0 ± 0.6	3.5

## Data Availability

The data that supports the findings of this study are available from the corresponding author on reasonable request.
